# Development and Application of a Multiple Cross Displacement Amplification Combined With Nanoparticle-Based Lateral Flow Biosensor Assay to Detect *Candida tropicalis*

**DOI:** 10.3389/fmicb.2021.681488

**Published:** 2021-06-10

**Authors:** Yu Wang, Xue Zhao, Jinzhi Cheng, Xiaomin Tang, Xu Chen, Honglan Yu, Shijun Li

**Affiliations:** ^1^Department of Clinical Laboratory, The First People’s Hospital of Guiyang, Guiyang, China; ^2^Laboratory of Bacterial Infectious Disease of Experimental Center, Guizhou Provincial Centre for Disease Control and Prevention, Guiyang, China; ^3^School of Basic Medical Sciences, Guizhou Medical University, Guiyang, China; ^4^Central Laboratory of the Second Affiliated Hospital, Guizhou University of Traditional Chinese Medicine, Guiyang, China

**Keywords:** *Candida tropicalis*, multiple cross displacement amplification, lateral flow biosensor, limit of detection, clinical samples

## Abstract

*Candida tropicalis* is an increasingly opportunistic pathogen that causes serious invasive candidiasis threatening a patient’s life. Traditional methods to detect *C. tropicalis* infection depends on time-consuming, culture-based gold-standard methods. So, we sought to establish a new method that could detect target pathogens quickly, accurately, and straightforwardly. Herein, a combination of multiple cross displacement amplification (MCDA) and lateral flow biosensors (LFB) was employed to detect *C. tropicalis.* In the MCDA system, 10 primers were designed to identify the specific genes of *C. tropicalis* and amplify the genes in an isothermal amplification device. Then, MCDA amplification reaction products could be identified visibly by color change, and all the amplification products would be tested by LFB with no special equipment. The results demonstrated that the optimal reaction condition of *C. tropicalis*-MCDA assay was 64°C within 30 min, and only 10 fg DNA was required in each reaction. No cross-reaction was found between *C. tropicalis* strains and non-*C. tropicalis* strains. For 300 sputum samples, the results showed that MCDA-LFB assay could rapidly and successfully detect all of the *C. tropicalis*-positive (28/300) samples detected by the gold-standard method. The entire procedure, including specimen processing (40 min), isothermal reaction (30 min) and result reporting (within 2 min), could be completed within 75 min. Briefly, the study results demonstrated that the detection ability of *C. tropicalis-*MCDA-LFB assay was better than culture methods with more simplicity, rapidity, sensitivity and specificity. Hence, MCDA-LFB strategy is an effective tool to rapidly detect *C. tropicalis* in clinical samples, especially in resource-poor areas.

## Introduction

Invasive candidiasis is a well-known life-threatening disease and a major issue for a medical institution, leading to significant morbidity, mortality, and huge extra hospital costs (Fuchs et al., 2019; Jenks et al., 2020). Regarding epidemiological data, invasive candidiasis was ranked the fourth leading nosocomial infectious disease with an overall mortality rate of 40% (McCarty and Pappas, 2015). Although *Candida albicans* is the most common species cause of invasive candidiasis, *C. tropicalis* has become an ascendent non-albicans *Candida* species causing invasive candidiasis worldwide, and its prevalence varies across geographic regions (Jagdish et al., 2013; Arrua et al., 2015; Chakrabarti et al., 2015). Compared to other non-*C. tropicalis Candida* species, *C. tropicalis* is stated to be more clinically indistinguishable and differently resistant to antifungal drugs. Besides, some previous studies indicated that invasive candidiasis caused by *C. tropicalis* have higher mortality compared to those caused by other non-*tropicalis Candida* species (Wang et al., 2016a; Fan et al., 2017). *C. tropicalis* is a major opportunistic human pathogenic yeast that is prevalent in nature and can be found in the skin, vagina, mouth and digestive tract of healthy people (Patil et al., 2018; Wang Q. et al., 2020). When patients with low immunity, especially those with neutropenia, leukemia, tumors, and bone marrow transplants are susceptible to *C. tropicalis*, this causes various clinically relevant infections, including candidiasis and systemic disseminated infections resulting in significant morbidity and mortality (Wang et al., 2016b; Horváth et al., 2020).

While antifungal drugs can be employed to treat *Candida* infections, mortality rates continue to rise (Ala-Houhala et al., 2019; Arastehfar et al., 2020). Furthermore, the epidemiology change of invasive candidiasis is partly attributed to overusing antifungal drugs, and some studies suggested that *C. tropicalis* appears to be resistant to azole antifungals, especially fluconazole, compared to other *Candida* species (Chakrabarti et al., 2009; Xiao et al., 2015; Chen et al., 2019). With the emergence of antifungal resistance and epidemiology change among *Candida* species, rapid and accurate fungi detection is required to optimize antifungal therapy.

So far, *C. tropicalis* can be detected by the commonly used culture and several non-culture methods (Chai et al., 2010). However, 3–7 days are needed to isolate and identify, *C. tropicalis* isolates using conventional culture method, which is considered the gold-standard method. In order to improve the detection duration and sensitiveness of laboratory diagnosis. Several non-culture methods have been developed for *C. tropicalis* detection, including polymerase-chain-reaction (PCR) assays and real-time PCR (Ogata et al., 2015; Guo et al., 2016). However, these methods require expensive laboratory equipment and highly skilled professionals, which are not available in resource-limited settings (Avni et al., 2011). On the other hand, microscopic examination is inaccurate, and traditional culture-based methods require at least 72 h, leading to optimal treatment time loss. Compared with other methods, multiple cross displacement amplification (MCDA) is a novel nucleic acid amplification technique under isothermal conditions, which avoid many complicated steps and expensive instruments (Wang et al., 2015). Besides, it only takes 1 h to complete gene amplification with color change (Wang J. et al., 2020). The amplification products can be tested using gold nanoparticle-based lateral flow biosensors (LFB), a new method to detect microbial specific gene fragments (Wang et al., 2016c).

Herein, we aim to develop a rapid and straightforward MCDA-LFB assay using specific primers targeting the internal transcribed spacer II (ITS II) gene of *C. tropicalis* for detecting *C. tropicalis* strains. The optimal conditions, analytical sensitivity, specificity, and feasibility of this method were validated utilizing pure cultures and clinical samples.

## Materials and Methods

### Reagents and Instruments

The kit Qiagen QIAamp DNA mini kit (Qiagen, Beijing, China) was used to extract genomic DNA from fungi, bacterial and clinical. Isothermal amplification kits and colorimetric indicator (malachite green, MG) were provided by Beijing-Hai Tai Zheng Yuan Technology Co., Ltd. (Beijing, China). Dye streptavidin-coated polymer nanoparticles (Crimson red) were obtained from Bangs Laboratories, Inc. (Indiana, United States). Anti-FAM (rabbit anti-fluorescein antibody) and biotin-BSA (biotinylated bovine serum albumin) were obtained from Abcam. Co., Ltd. (Shanghai, China). Polymer nanoparticle-based lateral flow biosensor (LFB) materials, including sample pad, nitrocellulose membrane (NC), absorbent pad, conjugate pad and backing card were obtained from Jie-Yi Biotechnology. Co., Ltd. (Shanghai, China). Primers and labeled primers (Table 2 and Supplementary Table 1) used were synthesized by Beijing-Tsingke Biotechnology Co., Ltd. (Beijing, China).

### Fungi, Bacteria Strains, Clinical Specimens, and Genomic DNA Preparation

A total of 37 fungi strains and 5 bacterial strains were utilized (Table 1), including 23 *C. tropicalis* and 14 non-*C. tropicalis* fungi strains. Except for *C. tropicalis* standard strain (ATCC13803) and *C. albicans* strain (ATCC10231), all the other strains were isolated from *The First People’s Hospital of Guiyang*, identified by culture and biochemical methods. All pure strains with 15% (w/v) glycerol broth were stored at −70°C. *Candida* strains were inoculated in the sabouraud dextrose agar (SDA) plate at 30°C, while bacteria strains were inoculated in the nutrient agar plate at 36°C. After 48 h of pure culture, all colonies were collected separately. To test the applicability of the *C. tropicalis* MCDA-LFB assay to clinical samples, 300 sputum samples were collected from June 1st to October 1st 2020 in the clinical microbiology laboratory, *The First People’s Hospital of Guiyang*. The protocol was approved by the Ethics Committee of *The First People’s Hospital of Guiyang*. Patients who provided sputum samples gave written informed consent, in accordance with the Declaration of Helsinki.

**TABLE 1:** Fungi and bacteria strains used in the current study.

Strains	Strain no. (source of strain)^a^	No. of strains	*Candida tropicalis-MCDA-LFB^b^*
*Candida tropicalis*	ATCC13803	1	P
*Candida tropicalis*	Isolated strains (GFPH)	23	P
*Candida albicans*	Isolated strains (GFPH)	3	N
*Candida albicans*	ATCC10231	1	N
*Candida dubliniensis*	Isolated strains (GFPH)	1	N
*Candida stellatoidea*	Isolated strains (GFPH)	1	N
*Candida guilliermondi*	Isolated strains (GFPH)	1	N
*Candida krusei*	Isolated strains (GFPH)	1	N
*Candida parapsilosis*	Isolated strains (GFPH)	1	N
*Candida glabrata*	Isolated strains (GFPH)	1	N
*Cryptococcus neoformans*	Isolated strains (GFPH)	1	N
*Penicillium mameffei*	Isolated strains (GFPH)	1	N
*Aspergillus flavus*	Isolated strains (GFPH)	1	N
*Staphylococcus aureus*	Isolated strains (GFPH)	1	N
*Enterococcus faecium*	Isolated strains (GFPH)	1	N
*Klebsiella pneumoniae*	Isolated strains (GFPH)	1	N
*Pseudomonas aeruginosa*	Isolated strains (GFPH)	1	N
*Acinetobacter baumannii*	Isolated strains (GFPH)	1	N

*^a^GFPH, The First People’s Hospital of Guiyang; ATCC, American Type Culture Collection.*

*^b^P, positive; N, negative. Only genomic DNA templates from *C. tropicalis* could be detected by MCDA-LFB assay, indicating the extremely high specificity of the method.*

DNA was extracted from each strain using a QIAamp DNA extraction kit (Qiagen, Beijing, China) following manufacturer’s instructions, and concentration and purity were measured with a Nanodrop 2000 (Beijing, China) at A260/280. DNA from *C. tropicalis* (ATCC13803) was employed as a positive control to confirm performance and choose optimal temperature and sensitivity, while DNA from *C. albicans* (ATCC10231) was deployed as a negative control. For clinical samples, DNA was extracted from 200 μL of the sputum sample using the QIAamp DNA extraction kit (Qiagen,Beijing,China) according to the manufacturer’s instructions. DNA was eluted by adding 60 μL of sterile, nuclease-free water. The extracted DNA were stored at −20°C until use.

### MCDA Assay Primers Design and Synthesis

**FIGURE 1:**
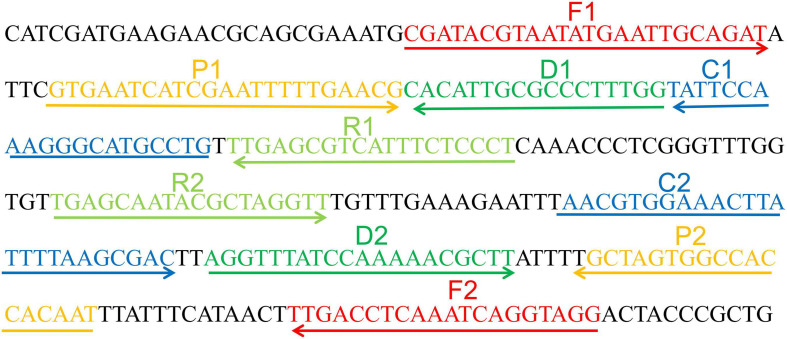
Appropriate primer design for *C. tropicalis* MCDA-LFB assay and primer sequence positions in the selected fragment including ITS II.

Primer Explorer V4 and Primer 5.0 were utilized to design MCDA primers targeting *C. tropicalis* specific gene ITS II (Accession No. AF268095.1). Based on design principle of MCDA primer, 10 primers used for *C. tropicalis* detection were designed and then blasted on NCBI to confirm their specificity (Wang et al., 2015). The details of MCDA primers’ locations and sequences were indicated in Figure 1 and Table 2. All of the primers used were synthesized by Beijing-Tsingke Biotechnology Co., Ltd. (Beijing, China) with HPLC purification grade.

**TABLE 2:** The primers used in the current study.

Primer name^*a*^	Sequences and modifications	Length^*b*^	Gene
F1	CGATACGTAATATGAATTGCAGAT	24 nt	ITS II
F2	CCTACCTGATTTGAGGTCAA	20 nt	
CP1	CAGGCATGCCCTTTGGAATAGTGAATCATC GAATTTTTGAACG	43 mer	
CP2	AACGTGGAAACTTATTTTAAGCGACATTGTG GTGGCCACTAGC	43 mer	
C1	CAGGCATGCCCTTTGGAATA	20 nt	
C1*	Biotin-CAGGCATGCCCTTTGGAATA	20 nt	
C2	AACGTGGAAACTTATTTTAAGCGAC	25 nt	
D1	CCAAAGGGCGCAATGTG	17 nt	
D1*	Fam-CCAAAGGGCGCAATGTG	17 nt	
D2	AGGTTTATCCAAAAACGCTT	20 nt	
R1	AGGGAGAAATGACGCTCAA	19 nt	
R2	TGAGCAATACGCTAGGTT	18 nt	

^a^C1*, 5′-labeled with biotin when used in the MCDA-LFB assay; D1*, 5′-labeled with FAM when used in the MCDA-LFB assay.

*^b^mer, monomeric unit; nt, nucleotide.*

### Preparation of Nanoparticle-Based Lateral Flow Biosensor

Lateral flow biosensor (LFB) used in this study was prepared according to previously reported (Wang et al., 2015). In short, the biosensor was composed of four sections, including conjugate pad, sample pad, NC membrane, and absorbent pad. Dye streptavidin-coated polymer nanoparticles were laminated in the conjugate pad. Anti-FAM (0.25 mg/ml) and biotin-BSA (2.5 mg/ml) were conjugated onto the NC membrane for test line (TL) and control line (CL), respectively. The prepared LFB were preserved with a desiccant gel at room temperature.

### The *C. tropicalis-*MCDA Reaction

Based on the standard MCDA reaction (Wang et al., 2015), the total volume of MCDA reaction system was 25 μL, containing 0.4 μM each of displacement primers (F1 and F2), 0.8 μM each of amplification primers (C1^∗^, C2, R1, R2, D1^∗^, and D2), 1.6 μM each of cross primers (CP1 and CP2), 12.5 μL of 2 × reaction mix (Hai Tai Zheng Yuan, Beijing, China), 1.25 μL (10U) *Bst* DNA polymerase (Hai Tai Zheng Yuan, Beijing, China) and 1 μL DNA template from isolated strain or 5 μL DNA template from clinical samples were used. Also, 10 ng genomic DNA of *C. albicans* ATCC10231 and *Klebsiella pneumoniae* was used as negative control, and 1 μL double distilled water was served as blank control.

Both colorimetric indicator (malachite green, MG) and lateral flow biosensor (LFB) methods were employed to determine and verify the *C. tropicalis*-MCDA products. When using MG, noticeable change was observed in the positive reaction solution from colorless to light green, while the negative and blank controls remained colorless. With LFB assay, two lines included test line (TL) and control line (CL), which appeared in positive reactions, but only CL was observed in negative controls and blank control.

### Optimal Temperature of *C. tropicalis-*MCDA Assay

In the MCDA reaction system, temperature was essential for influencing amplification efficiency. the amplification temperature of *C. tropicalis*-MCDA assay was optimized from 61 to 68°C with 1°C interval. Amplification mixtures with 1 μL of template of *C. albicans* were used as negative controls (NCs), and 1 μL of distilled water (DW) was used as a blank control. The MCDA amplicons were monitored using loopamp real-time turbidimeter LA-320C (Eiken Chemical Co., Ltd., Japan). Turbidity >0.1 was considered as positive result.

### Limit of Detection (LoD) and Optimal Isothermal Amplification Time of *C. tropicalis-*MCDA Assay

To evaluate LoD of MCDA assay for *C. tropicalis*, a serial dilution of *C. tropicalis* strain (ATCC13803) genomic DNA from 10 ng to 100 ag (10 ng, 10 pg, 1 pg, 100 fg, 10 fg, 1 fg, and 100 ag per microliter) were used to determine the LoD. As a template, 1 μL genomic DNA was added into the amplification reaction system. Meanwhile, the serially diluted templates were applied for optimizing the isothermal amplification time during the reaction stage, four different times (10–40 min, with 10 min interval) were compared under the same reaction conditions, and all of the amplified products at each time point including 10, 20, 30, and 40 min were detected by MG and LFB, and each reaction was repeated three times.

### Specificity of *C. tropicalis-*MCDA Assay

To assess the analytical specificity of *C. tropicalis*-MCDA assay, the genomic DNA (at least 10 fg per microliters) was extracted from 23 *C. tropicalis* strains and 18 non-*C. tropicalis* strains, respectively, which were amplified under the best conditions. All MCDA products were detected by lateral flow biosensor (LFB), and each test was repeated at least three times.

### Application of *C. tropicalis -*MCDA Detection in Clinical Samples

To evaluate applying *C. tropicalis*-MCDA detection in clinical samples, 300 sputum samples were collected from *The First People’s Hospital of Guiyang* and were detected using the gold-standard method (the culture method) and *C. tropicalis*-MCDA. Traditional culture methods included colony morphology, Gram staining and biochemical identification. The traditional detection method was performed first, MCDA-LFB was performed after a certain amount of sputum samples were collected, culture and molecular testing were done by two different people, and it was not known which one contained the target fungus before the experiment. The *C. tropicalis*-MCDA detection was performed as described above. The results of *C. tropicalis*-MCDA-LFB were compared with that of culture assay.

## Results

### Selection of the Set of Primers

**FIGURE 2:**
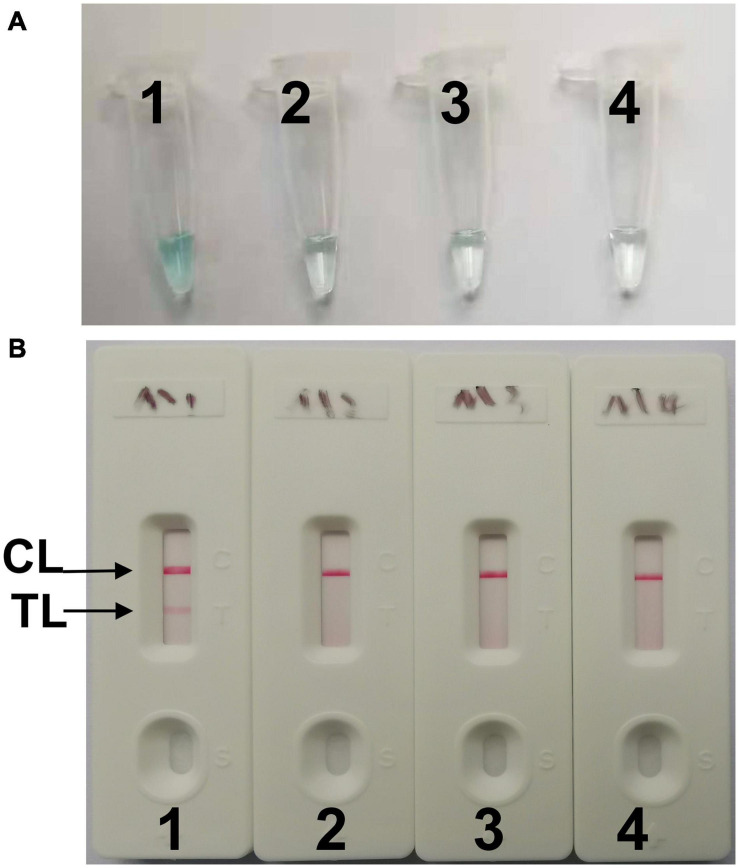
Confirming of *C. tropicalis*-MCDA products. **(A)** The *C. tropicalis*-MCDA amplification products were analyzed with MG reagents through visually observation of the color change. **(B)** Lateral flow biosensor (LFB) was applied for visual detection of *C. tropicaliss*-MCDA products, two lines contained test line (TL) and the control line (CL) appeared in positive samples, but only the CL observed in negative samples. Tube 1/Biosensor 1: positive amplification of *C. tropicalis* (ATCC13803); Tube 2/Biosensor 2: negative amplification of *C. albicans* (ATCC10231); 3: negative amplification of *Klebsiella pneumoniae*; Tube 4/Biosensor 4: blank control (DW).

To confirm effectiveness of the four sets of primers (Supplementary Table 1) for *C. tropicalis*, MCDA reactions were performed at a constant temperature of 62°C for 40 min using *C. tropicalis* (ATCC13803) genomic DNA templates. The most effective set of primers was selected by inspection of the curves from loopamp real-time turbidimeter LA-320C for use as future experimental primers (Supplementary Figure 1). The first set primers is considered to be the most effective for *C. tropicalis*-MCDA amplification, because a threshold value of 0.1 of absorbance that indicated positive amplification from the *C. tropicalis*-MCDA reaction was reached most quickly. Then, to confirm the effectiveness of the MCDA primers we selected (Figure 1 and Table 1), the amplification products were monitored by two different methods, including colorimetric indicator (malachite green, MG) and lateral flow biosensor (LFB). The results showed that the positive results were observed when the nucleic acid from *C. tropicalis*, but not with *C. albicans*, *Klebsiella pneumoniae*, and the blank control (Figures 2A,B). Hence, the *C. tropicalis*-MCDA primers for ITS II gene detection in the current study were valid for the establishment of *C. tropicalis* MCDA-LFB.

### Optimal Temperature of *C. tropicalis-*MCDA Assay

**FIGURE 3:**
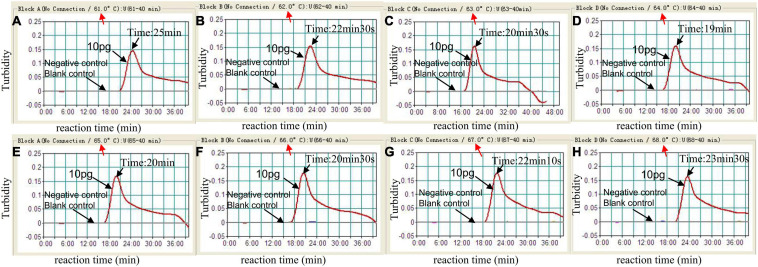
Optimal amplification temperature for *C. tropicalis*-MCDA assay. By using a real-time measurement to monitor the turbidity of *C. tropicalis*-MCDA reactions. The corresponding curves were displayed in the panels. The negative control was *C. albicans*, and the blank control was sterile double-distilled water. Abscissa represents reaction time (min), ordinate represents turbidity. The threshold value was 0.1, and the turbidity >0.1 was considered as positive amplification. Eight kinetic curves **(A–H)** were generated from 61 to 68°C (1°C intervals), with 10 pg *C. tropicalis* DNA per reaction. The optimal *C. tropicalis*-MCDA reaction temperature was 64°C.

To identify the optimal temperature of *C. tropicalis*-MCDA assay, the *C. tropicalis* standard strain (ATCC13803) DNA was employed as a template, and MCDA amplifications were carried out from 61 to 68°C with 10 pg per tube of *C. tropicalis.* The turbidity of all reaction products was monitored using loopamp real-time turbidimeter LA-320C. As shown in Figure 3, 64°C was considered as an optimum reaction temperature for *C. tropicalis*-MCDA amplification, because a threshold value of 0.1 of absorbance that indicated positive amplification from the *C. tropicalis*-MCDA reaction was reached most quickly at 64°C. Herein, the optimal temperature of 64°C was employed for the subsequent *C. tropicalis-*MCDA examinations conducted in this report.

### Limit of Detection and Optimized Time of MCDA for *C. tropicalis* Detection

**FIGURE 4:**
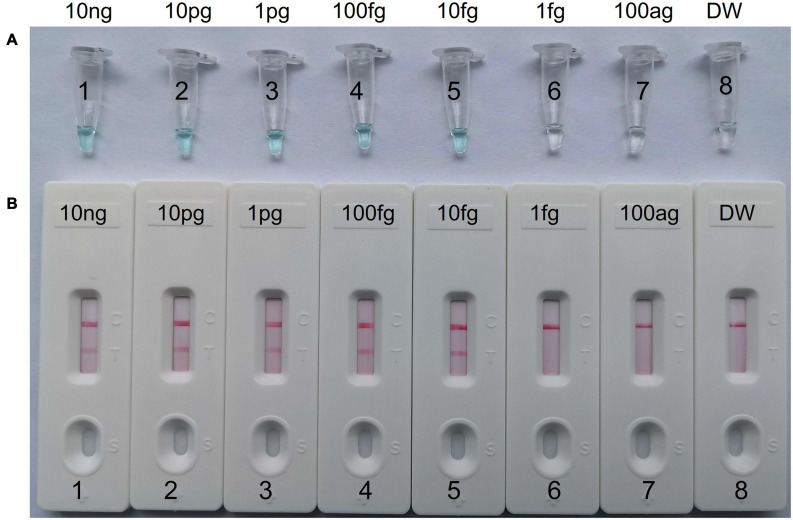
The detection limit of *C. tropicalis*-MCDA-LFB assays. Two measurement techniques, including **(A)** a colorimetric indicator (MG) and **(B)** lateral flow biosensor. A series of dilutions (10 ng, 10 pg, 1 pg, 100 fg, 10 fg, 1 fg, and 100 ag) of *C. tropicalis* ATCC13803 DNA and a blank control (DW) were operated according to standard MCDA reactions.

To determine the LoD of *C. tropicalis*-MCDA assay, a serial dilution of the *C. tropicalis* genomic DNA (10 ng, 10 pg, 1 pg, 100 fg, 10 fg, 1 fg, and 100 ag per microliter) was used in MCDA assays. As observed in Figure 4, when the dilution exceeded 10 fg, MCDA tubes presented colorless and only control band in LFB. While other dilutions MCDA tubes were presented bright blue, and two red bands were observed in LFB; one was control line, and the other was test line.

**FIGURE 5:**
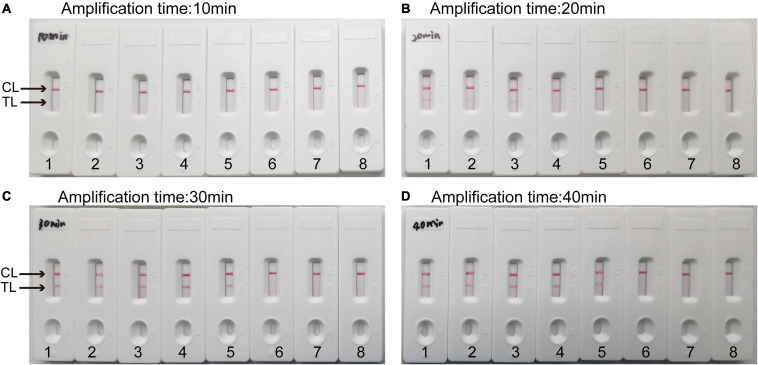
Optimal detection time required for *C. tropicalis* MCDA-LFB assay. Four different reaction times (**(A)** 10 min; **(B)** 20 min; **(C)** 30 min; **(D)** 40 min) were evaluated at 64°C. Biosensors 1–7 represent *C. tropicalis* ATCC13803 DNA levels of 10 ng, 10 pg, 1 pg, 100 fg, 10 fg, 1 fg, and 100 ag per reaction, respectively; 8 represents a blank control (DW). The best amplification time was observed when MCDA lasted for 30 min **(C)**.

To obtain the optimal amplification time for the *C. tropicalis* MCDA assay, four amplification times (10, 20, 30, and 40 min) were tested at 64°C, respectively. The amplification products were tested with LFB. The results confirmed that the LoD level of *C. tropicalis* ATCC10231 genomic DNA (10 fg per reaction) was tested when the amplification last 30 and 40 min (Figure 5). Therefore, 30 min was considered an appropriate amplification time for *C. tropicalis* MCDA assay.

### Specificity of MCDA-LFB for *C. tropicalis* Detection

**FIGURE 6:**
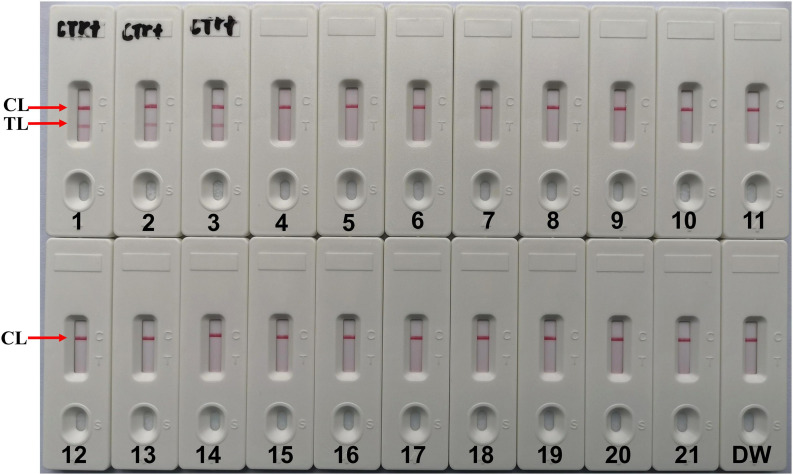
Specificity of LFB assays detecting *C. tropicalis*-MCDA products. The *C. tropicalis*-MCDA-LFB assay was evaluated with different genomic DNA as templates. Both the control line and the test line were visible in LFB for all *C. tropicalis*, and only the control line was appeared in non- *C. tropicalis*. LFB devices were labeled *C. tropicalis* (Ctr) as positives after the test results were obtained. 1, Positive control (*C. tropicalis* ATCC13803), 2–3, isolated *C. tropicalis* strains; 4–6, *Candida albicans*; 7, *Candida dubliniensis*; 8, *Candida stellatoidea*; 9, *Candida guilliermondi*; 10, *Candida krusei*; 11–12, *Candida parapsilosis*; 13, *Candida glabrata*; 14, *Cryptococcus neoformans*; 15, *Penicillium mameffei*; 16, *Aspergillus flavus*; 17, *Staphylococcus aureus*; 18, *Enterococcus faecium*; 19, *Klebsiella pneumoniae*; 20, *Pseudomonas aeruginosa*; 21, *Acinetobacter baumannii*; 22, a blank control (DW).

The results showed that two red lines appeared at the location of TL and CL on the strips for the *C. tropicalis* strain, but only one line appeared at the location of CL for all the non-*C. tropicalis* strains and blank control (Figure 6), LFB devices were labeled *C. tropicalis* (Ctr) as positives after the test results were obtained. So, the results suggested that MCDA-LFB was highly specific for *C. tropicalis* detection.

### Application of MCDA-LFB Assay in Clinical Samples for *C. tropicalis* Detection

To further confirm the application of MCDA-LFB as a valuable tool for *C. tropicalis* detection in clinic, we analyzed 300 sputum samples from clinic using culture methods and *C. tropicalis-*MCDA-LFB assay. As *C. tropicalis* positive bloodstream samples are not frequently obtained in our clinic, but positive sputum samples could be readily obtained (Xie et al., 2019), sputum samples were chosen to investigate the clinical utility of the test. The results are shown in Table 3. Of the 300 clinical sputum samples, 28 sputum samples were detected successfully by MCDA-LFB assay, which had been confirmed positive by culture assays. The *C. tropicalis-*MCDA-LFB assay results were the same as traditional culture results. Therefore, such results suggested that MCDA-LFB was a feasible alternative to traditional culture and could potentially provide results in nucleic acid analysis.

**TABLE 3:** Comparison of methods for detection of *C. tropicalis* in sputum samples.

Detection methods	Sputum samples (*n* = 300)	
	
	Positive	Negative
Culture-based assay	28	272
MCDA-LFB assay	28	272

## Discussion

*C. tropicalis* is a diploid dimorphic opportunistic fungus belonging to a member of genus *Candida*. *C. tropicalis* has recently become one of the most common non-*Candida albicans Candida* species (NAC), even more prevalent than *C. albicans* in some countries. It is reported that non-*Candida albicans Candida* species are clinically indistinguishable and displays different resistance to antifungal drugs and degrees of virulence (Fan et al., 2017). Furthermore, mortality rates associated with non-*Candida albicans Candida* species infection are unacceptably high (40–70%), especially patients with immunosuppressed and neutropenic or bone marrow transplant (Xiao et al., 2020). So rapid diagnosis and accurate medication are key to improving the survival rate. However, traditional methods (culture-based techniques and colony morphology) and PCR-based technology for detecting *C. tropicalis* are time-consuming. Consequently, developing a new method to identify and diagnose *C. tropicalis* straightforwardly, rapidly, sensitively, and specifically is required.

This study combined multiple cross displacement amplification (MCDA) with lateral flow biosensors (LFB) to detect *C. tropicalis.* The MCDA is an isothermal amplification system that requires only a simple water bath or heater without special equipment and professional training (Wang et al., 2017). The LFB labeled with FAM and biotin could detect amplification products (Wang et al., 2018). We could read the test result from the lines on LFB for approximately 2 min. Compared to molecular diagnostic assays, such as PCR-based methods, 10 primers were designed to identify the specific genes of *C. tropicalis* in MCDA system, and the study results showed that the optimal reaction condition of *C. tropicalis*-MCDA assay was 64°C within 30 min, and only 10 fg DNA was required in each reaction. This indicates that MCDA is a rapid, sensitive and straightforward method for detecting trace amounts of *C. tropicalis*. The specificity of *C. tropicalis*-MCDA-LFB assay was confirmed using the template DNA isolated from 23 *C. tropicalis* strains and 18 non-*C. tropicalis* strains, and all *C. tropicalis*-positive products from MCDA were successfully identified using LFB, not for non-*C. tropicalis* strains, which indicated that *C. tropicalis*-MCDA-LFB assay could be regarded as an applied tool for detecting *C. tropicalis* with high specificity. The entire detection procedure of *C. tropicalis*-MCDA-LFB assay, including template preparation (about 30 min), isothermal reaction (30 min), and LFB result reading (approximately 2 min), could be performed within 70 min. Though MCDA amplification products could be detected by gel electrophoresis analysis and turbidity analysis, we chose LFB in this study. In the detection procedure, using LFB could avoid many tedious steps such as gel electrophoresis analysis, and LFB result reading does not require professional training. Furthermore, we can obtain experiment result only by waiting for approximately 2 min, implying that LFB is easier and shortens the detection time than other methods.

More importantly, we successfully used *C. tropicalis*-MCDA-LFB assay to detect sputum samples from clinic. Compared with culture-based method, MCDA-LFB method is more rapid and straightforward, with 100% positive rate and 100% negative rate. Compared with real-time PCR assay for candida species, the *C. tropicalis*-MCDA-LFB technique is more time-saving, the real-time PCR requires 2∼3 h during the whole process (Guo et al., 2016). However, the detection time of entire MCDA-LFB assay procedure was less than 1.5 h, including sample processing. It showed that our developed method to detect *C. tropicalis* is faster than culture-based and real-time PCR method. Since *C. tropicalis*-MCDA-LFB assay can report results within only 1.5 h, clinicians can provide targeted therapies to patients more quickly, thus reducing exposure to broad-spectrum antibiotics.

However, our method has limitations. Firstly, the MCDA-LFB detection is a qualitative determination for *C. tropicalis*, and could not quantify target pathogen amount in the sample. so we cannot distinguish the infection from colonization. It is challenging to assess the dosage and efficacy of antibiotics. A more precise study will be designed for quantification of the amount of *C. tropicalis* in clinical samples with MCDA-LFB assay. Secondly, we only use the method to detect clinical sputum samples, and other specimens that can be tested in this way are unknown. In recent years, the proportion of bloodstream infection and urinary tract infection caused by *C. tropicalis* has increased, especially in intensive care unit patients, especially in those with malignancies, receiving broad-spectrum antibiotics or undergoing prolonged catheterization (Gharanfoli et al., 2019; Wang J. et al., 2020). So, we will focus on testing other clinical samples such as blood and urine in MCDA-LFB method. Moreover, MCDA is an isothermal amplification system requiring several multiple pairs of primers that easily carry contamination leading to false-positive results. Efforts have been made to solve this problem, and if we hold the optimum reaction temperature and reaction time, we can reduce false-positive results. So far, the MCDA-LFB assay has been successfully used to detect pathogens such as *Staphylococcus aureus*, *Acinetobacter baumannii*, *Shigella* spp., and *Candida albicans* (Wang et al., 2016d, 2018; Cheng et al., 2019; Zhao et al., 2019).

## Conclusion

In this work, we successfully developed an MCDA-LFB assay to detect *C. tropicalis* simply, rapidly, and precisely. Compared with culture-based methods and molecular diagnostic assays, MCDA-LFB assay avoids sophisticated processes and does not require expensive equipment and skilled technical personnel. The limit of detection (LoD) of novel assay for *C. tropicalis* detection from isolate was as little as 10 fg, suggesting that MCDA-LFB assay was very sensitive. More importantly, the novel assay is more time-saving that can reduce detection time, and help clinicians provide targeted therapies for patients more quickly. In short, MCDA-LFB assay with accurate and timely detection, and not require expensive equipment and skilled technical personnel, it is amenable to generalize, especially in resource-poor areas.

## Data Availability Statement

The original contributions presented in the study are included in the article/Supplementary Material, further inquiries can be directed to the corresponding author/s.

## Ethics Statement

Written informed consent was obtained from the minor(s)’ legal guardian/next of kin for the publication of any potentially identifiable images or data included in this article.

## Author Contributions

YW, XT, and SL conceived and designed the experiments. YW, XZ, JC, XT, and XC performed the experiments. YW, JC, and HY analyzed the data. YW, XZ, XC, and SL wrote the manuscript. All authors contributed to the article and approved the submitted version.

## Conflict of Interest

The authors declare that the research was conducted in the absence of any commercial or financial relationships that could be construed as a potential conflict of interest.
