# Supplementary material for: Development and Application of a Multiple Cross Displacement Amplification Combined With Nanoparticle-Based Lateral Flow Biosensor Assay to Detect Candida tropicalis
Source: Front Microbiol. 2021 Jun 10;12:681488. doi: 10.3389/fmicb.2021.681488 (PMC8222920; doi:10.3389/fmicb.2021.681488)
Supplement: Supplementary file 1 [file Data_Sheet_1.PDF]

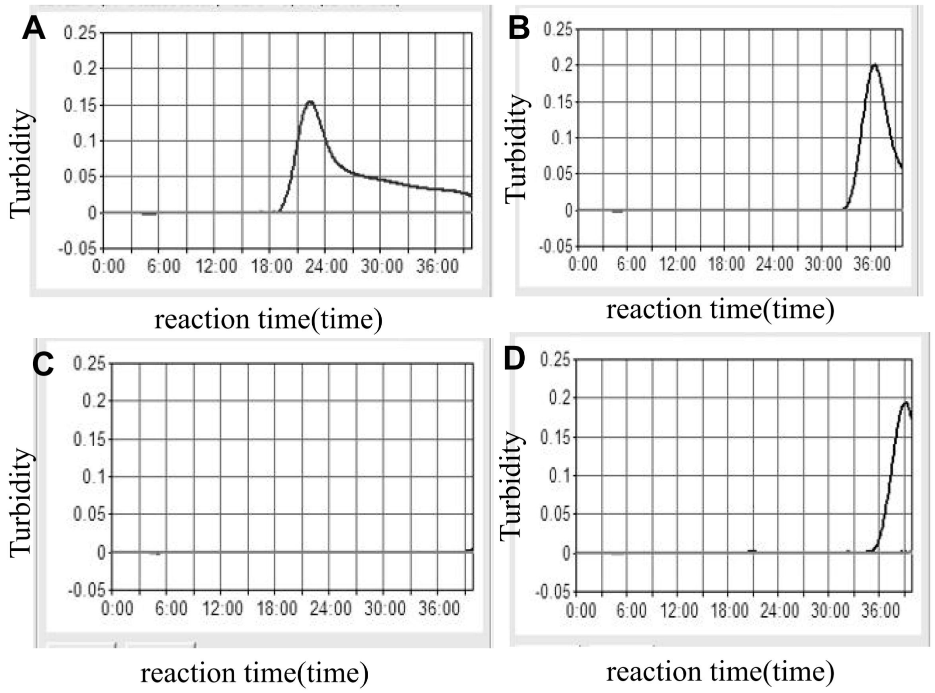

Figure S1. The four sets of primers (Table S1) for *C. tropicalis*-MCDA reactions.A.The first set of primers; B.The second set of primers; C.The third set of primers; D. The fourth set of primers. The threshold value was 0.1, and the turbidity > 0.1 was considered as positive amplification.
